# A comparative analysis of transcranial Doppler parameters acquired during carotid stenting and semi-eversion carotid endarterectomy

**DOI:** 10.1590/1677-5449.003316

**Published:** 2016

**Authors:** Germano da Paz Olveira, Ana Terezinha Guillaumon, Sérgio Clementino Benvindo, Joana Mayra Teixeira Lima, Sérgio Ricardo Freire Barreto, Wagner Mauad Avelar, Fernando Cendes

**Affiliations:** 1 Universidade Estadual de Campinas – UNICAMP, Departamento de Cirurgia, Campinas, SP, Brazil.; 2 Centro Universitário Uninovafapi, Teresina, PI, Brazil.; 3 Universidade Federal do Piauí – UFPI, Teresina, PI, Brazil.; 4 Universidade Estadual de Campinas – UNICAMP, Departamento de Neurologia, Campinas, SP, Brazil.

**Keywords:** carotid stenosis, carotid endarterectomy, transcranial Doppler ultrasonography, estenose das carótidas, endarterectomia das carótidas, ultrassonografia Doppler transcraniana

## Abstract

**Background:**

Carotid endarterectomy (CEA) and carotid artery stenting (CAS) have both been proposed for treatment of critical atherosclerotic stenosis located at the carotid bifurcation. Monitoring of hyperintense microembolic signals (MES) by transcranial Doppler ultrasound (TCD) is considered a method of quality control, both in CEA and in CAS.

**Objective:**

To analyze temporal distribution of MES throughout both semi-eversion CEA and CAS procedures and to evaluate changes in mean velocity of blood flow through the ipsilateral middle cerebral artery (MCA).

**Method:**

Thirty-three procedures (17 CEA and 16 CAS) were prospectively monitored using TCD and the data were related to three different stages of surgery (pre-cerebral protection, during cerebral protection and post-cerebral protection). Chi-square, Mann-Whitney, ANOVA and contrast tests were used for statistical analysis.

**Results:**

The MES were uniformly distributed in the CEA group, but not in the CAS group (p = 0.208). The number of MES was higher in the CAS group in all stages. The average flow in the MCA was similarly lower in both groups during the protection stage.

**Conclusion:**

CEA provoked a lower incidence of MES per procedure than CAS in all stages. The behavior of the averages of the mean of blood flow through the MCA was similar in both groups.

## Resumo

**Contexto:**

A endarterectomia carotídea (EC) e a angioplastia carotídea (AC) são propostas para o tratamento de estenoses críticas localizadas na bifurcação carotídea. O monitoramento dos sinais de microembolias (SMs) pela ultrassonografia Doppler transcraniana (UDT) é considerado um método de controle de qualidade para ambas as técnicas.

**Objetivos:**

Analisar a distribuição temporal dos SMs ao longo de diferentes estágios da EC por semieversão e da AC, e avaliar o significado das mudanças nas médias das velocidades médias do fluxo na artéria cerebral média ipsilateral (ACM).

**Método:**

Trinta e três procedimentos (17 ECs e 16 ACs) foram monitorados com UDT, e os dados foram coletados prospectivamente para diferenciar os diferentes estágios cirúrgicos (pré, durante e pós-proteção cerebral). Para análise estatística foram usados os testes qui-quadrado, Mann-Whitney, análise de variância (ANOVA) e contraste.

**Resultados:**

Em ambos os grupos, os SMs foram distribuídos uniformemente (p = 0,208). Em todos os tempos, o número de SMs foi superior no grupo AC. A média das velocidades médias do fluxo na ACM foi menor durante o tempo de proteção em ambos os grupos.

**Conclusão:**

A EC teve uma menor incidência de SMs que a AC em todos os estágios. A média das velocidades médias na ACM teve comportamento similar em ambos os grupos.

## INTRODUCTION

Carotid endarterectomy (CEA) and carotid artery stenting (CAS) have both been proposed for treatment of critical atherosclerotic stenosis located at the carotid bifurcation. It has been scientifically accepted that CAS is increasingly an important alternative to CEA.^1^^-^ 3

Transcranial Doppler ultrasound (TCD) is a non-invasive technique that has been used during carotid revascularization with the objective of monitoring hemodynamic changes as well as for identifying hyperintense microembolic signals (MES) resulting from embolic materials.^4^

Some studies have reported that microemboli can predict cerebrovascular symptoms,^5^^-^ 7 but this has not been confirmed by others.^8^^,^ 9 There is evidence that these microemboli can also contribute to dementia.^10^^,^ 11 Monitoring of MES by TCD is considered a method for quality control, both in CEA and in CAS.^12^^,^ 13

This study aims to analyze the temporal distribution of MES throughout three different stages of both CEA (semi-eversion technique) and CAS and to evaluate changes in mean velocity of blood flow in the middle cerebral artery (MCA).

## METHODS

Institutional review board approval was obtained to prospectively analyze patients who would undergo elective carotid revascularization with TCD monitoring. The TCD endpoints were the number of ipsilateral MES generated during the procedure, its temporal distribution, and also the variation of the averages of mean velocity in the ipsilateral MCA. The procedures were divided into three stages: pre-cerebral protection (until the internal carotid artery [ICA] is clamped or a distal filter deployed), during cerebral protection (until antegrade flow in the ICA is re-established or the filter removed) and post-cerebral protection (after antegrade blood flow in the ICA is re-established or the filter removed). Cerebral protection time was measured in seconds and corresponded to the duration of the second stage. Another variable analyzed was the duration of ischemia, which was classified as the period of time during which the average velocity of blood flow within the MCA fell below 30 percent of the pre-procedural velocity.

### Patients

From January 2010 to January 2012, a total of 60 carotid revascularization procedures were performed at the Universidade Estadual de Campinas (UNICAMP) University Hospital. The study was approved by the UNICAMP ethics committee and all patients signed an informed consent form prior to all procedures. Therefore, this study was performed in accordance with the 1964 Declaration of Helsinki. Treatment for symptomatic disease was offered for stenosis greater than 70%, according to the North American Symptomatic Carotid Endarterectomy Trial (NASCET) criterion,^14^ while for asymptomatic patients, the cutoff was 80%, in accordance with Asymptomatic Carotid Atherosclerosis Study (ACAS) recommendations.^15^ The severity of carotid stenosis was determined by Duplex ultrasound (DUS) and confirmed by angiography or angiotomography.

Patients were excluded because of the following: absence of a bone window for TCD monitoring (12 patients); carotid restenosis (1 patient); combined procedures (3 patients); presence of concurrent stenosis in the target artery (5 patients); need for urgent intervention (3 patients); and severe renal insufficiency (Cr > 2.0) (3 patients). Thirty-three carotid revascularization procedures (17 CEA and 16 CAS) were conducted with TCD monitoring and the data were prospectively entered into a database.

Tables 1 and 2 summarize baseline patient characteristics broken down by method of carotid revascularization. Overall, 87.8% of the patients were male and their mean age was 71.2 years. Hypertension and smoking history (more than 20 pack-years, quit ≥ 1 year previously) were prevalent in both treatment groups and both treatment groups included symptomatic and asymptomatic patients.

**Table 1:** Clinical and epidemiologic data (categorical variables).

**Variables**	**CEA (n = 17)**	**CAS (n = 16)**	**Total (n = 33)**	***p-value***
Male	15 (88.2%)	14 (87.5%)	29 (87.8%)	1.000*
Diabetes	6 (35.3%)	9 (56.2%)	15 (45.4%)	0.226†
Smoking history < 1 year	3 (17.6%)	3 (18.7)	6 (18.2%)	1.000*
Hypertension	15 (88.2%)	12 (75%)	27 (81.8%)	0.398*
Dyslipidemia	7 (41.2%)	10 (62.5%)	17 (51.5%)	0.220†
Renal insufficiency	0 (0%)	1 (6.2%)	1 (3.0%)	-
Smoking history > 1 year	11 (64.7%)	11 (68.7%)	22 (66.7%)	0.805†
CAD	5 (29.4%)	11 (68.7%)	16 (48.5%)	0.023†
Right-sided lesion	10 (58.8%)	6 (37.5%)	16 (48.5%)	0.220†
Total contralateral obstruction	1 (5.9%)	4 (25%)	5 (15.1%)	0.192*
70-99% contralateral obstruction	1 (5.9%)	2 (12.5%)	3 (9.1%)	0.192*
Symptomatic	7 (41.2%)	6 (37.5%)	13 (39.4%)	0.829†
General anesthesia	16 (94.1%)	8 (50%)	24 (72.7%)	0.006*

CAD: coronary artery disease; CAS: carotid artery stenting; CEA: carotid endarterectomy.

* Fisher test;

† chi-square test.

**Table 2:** Clinical and epidemiologic data (numerical variables).

**Groups** **Variables**	**CEA (n = 17)**					**CAS (n = 16)**					**p-value** *
	**Mean**	**SD**	**Min**	**Med**	**Max**	**Mean**	**SD**	**Min**	**Med**	**Max**	
Age (years)	71.5	5.6	63	69	81	70.9	7.1	54	72.5	78	0.772
Protection time (s)	1016.5	343.7	540	1020	2160	881	309.2	393	799.5	1360	0.357
Number of MES	89.8	171.4	5	46	745	597.5	343.3	172	610.5	1640	<0.0001
MBP variation (mmHg)	22.6	8	10	23	35	21.1	10.7	5	21	40	0.563
Duration of ischemia (s)	0	0	0	0	0	33.8	83.4	0	0	312	-

CAS: carotid artery stenting; CEA: carotid endarterectomy; MBP: mean systemic arterial blood pressure; MES: microembolic signals; SD: standard deviation.

* Mann-Whitney test.

### Carotid revascularization procedures

Criteria for selection of type of procedure (CEA or CAS) were in accordance with American Heart Association and American Stroke Association guidelines.^1^ Carotid artery stenting was only considered for patients with: open heart surgery < 6 weeks; myocardial infarction < 4 weeks; angina CCS (Canadian Cardiac Score) class III/IV; chronic heart failure class III/IV; ejection fraction < 30%; abnormal cardiac stress test; chronic oxygen therapy; resting pO_2_ < 60%; forced expiratory volume in 1 second < 50% predicted; previous ipsilateral CEA; cervical radiation treatment; high cervical lesion (at least C2); lesion below the clavicle; contralateral laryngeal palsy.

All physicians were trained in vascular and endovascular surgery. The protocols for CEA and CAS were standardized for general anesthesia or local anesthesia (according to the anesthesiologist’s preferences) and intraoperative TCD monitoring.

### Carotid endarterectomy

Patients were treated with 200 mg of aspirin during the perioperative period. Plaques were endarterectomized using a standard semi-eversion technique performed by a vascular surgeon. This technique involves a longitudinal arteriotomy limited to the carotid bulb, removal of the plaque using eversion, and closure of the arteriotomy.^16^ The semi-eversion technique permits a smaller arteriotomy and, consequently, a shorter clamping time. A bovine pericardial patch was used in cases in which arteries had diameters smaller than 7 mm, which occurred in one patient, with the intention of reducing the total duration of the procedure and the rate of infections. The criteria for using a shunt in this study was a 70 percent reduction in baseline mean flow velocity in the MCA after internal carotid clamping, but none of the patients in this cohort required shunting. After intravenous administration of heparin (80-100 IU/kg), the internal, external, and common carotid arteries were occluded, in that order. Heparin was reversed selectively by the anesthesiologist using protamine.

It is worth mentioning the method of unclamping used in the study. The internal and external carotid arteries were unclamped before unclamping of the common carotid artery, to achieve a backward flush from the ICA. Next, the ICA was clamped and only then was the common carotid artery unclamped, allowing blood to flow into the external carotid artery. Finally, the ICA was unclamped.

### Carotid artery stenting^17^

Patients were treated with 200 mg of aspirin and 75 mg of clopidogrel during the perioperative period. Patients were heparinized with 80-100 UI/kg, an arch angiogram was performed and the target carotid was selectively cannulated, in all cases by femoral puncture and using a coaxial system comprising a long catheter (5 French) with Simons 2 or Multipurpose tip, and a long sheath (6 French) with a straight tip. Distal filters used were SpiderFx (EV3 Endovascular Inc, Plymouth, Minn). Seven-mm or 8-mm self-expanding stents were deployed and postdilated to 5 or 6 mm. Stents used included the Protégé RX (EV3 Endovascular Inc), an open-cell stent variety. Atropine was administered selectively. Residual stenosis of 20% was considered an acceptable result. Contrast was injected after stent deployment to enable control imaging and there were no findings suggestive of vasospasm.

### Transcranial Doppler ultrasound^18^

Before the procedure, patients were examined by mapping out the blood flow of the MCA to confirm the acoustic window, using a TCD Sonara-USA, a 2-channel device with a 2 MHz transducer. New measures were taken during the procedures. The TCD was fitted over the temporal bone above the zygomatic arch on the side of the target carotid. Microembolic signals were identified automatically and recorded on a hard drive, and were collected across a wide insonation gate set at 45 to 55 mm, with a sample size of 5 mm. Each stage of the procedure was timed. The number of MES and measures of the average velocity of blood flow in the MCA were obtained by analyzing these data after the procedure, excluding signals that were related to interference produced by the electric scalpel or contrast injection. These values were automatically recorded by the machine, which has been validated in previous reports.^17^^,^ 19

### Statistical analysis

Descriptive analysis consisted of tables of frequencies for categorical variables and measures of position and dispersion for numerical variables. The chi-squared test or (when necessary) Fisher’s exact test were employed to compare proportions and the Mann-Whitney test was used to compare numerical measures. Analysis of variance (ANOVA) for repeated measures with rank transformation was conducted, followed by contrast tests, in order to compare groups, procedural stages, and the interaction between them. The level of statistical significance used was α = 5%. All statistical calculations were performed using SAS for Windows (Statistical Analysis System, version 9.2.; SAS Institute Inc, 2002-2008, Cary, NC, USA).

## RESULTS

The majority of clinical epidemiological data did not differ statistically between groups, but the groups were different in terms of coronary artery disease (CAD) and type of anesthesia (Tables 1 and 2). One of the 33 patients suffered a stroke, followed by death, 25 days after the procedure. This patient had undergone CAS with local anesthesia. All of the other patients were successfully treated with carotid revascularization with no complications (such as stroke, myocardial infarction, or death). Doppler US of the neck revealed no stenosis at 30 days.

Analysis of the intraoperative TCD data shown in Table 2 reveals important differences in duration of ischemia. However although this could be relevant from a clinical point of view, in terms of statistics this difference cannot be shown because there was no variation in the CEA group. No conceptual ischemia (< 30% of baseline) occurred during the procedure to treat the patient who later died.

There was a statistically significant difference between the groups (p < 0.0001) for the number of MES detected (Table 2). There was a mean of 89.8 (± 171.4) microembolic signals per procedure in the CEA group, while the mean number in the CAS group was 597.5 (± 343.3). A total of 661 microembolic signals were detected during the procedure to treat the patient who eventually died. This patient was in the CAS group, in which the maximum number of signals was 1640.

**Figure 1:**
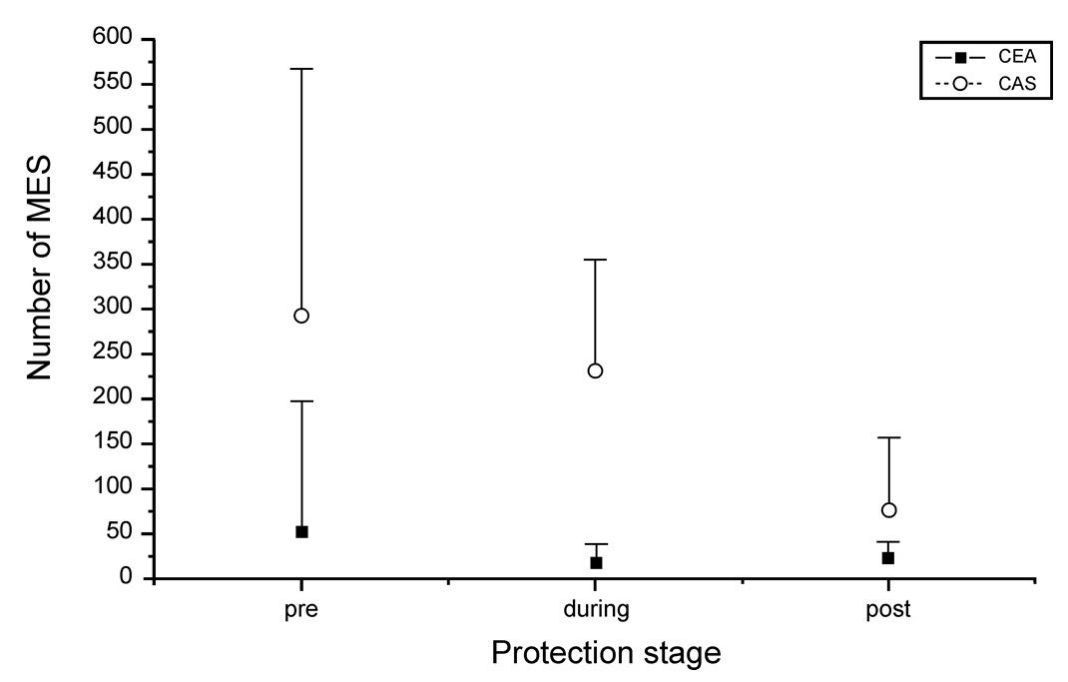
Average values with standard deviation of the number of MES in each protection stage.

The distribution of MES across the three stages of the procedures can be observed in Table 3 and is also illustrated in Figure 1. In the CEA group, the MES were uniformly distributed (p = 0.208). However, in the CAS group, notable differences (p < 0.0001) were detected between the pre-protection and post-protection stages and also between the during protection stage and the post-protection stage. No statistical differences were found between the first and second stages. In effect, the mean number of MES was higher in the CAS group in all three stages.

**Table 3:** Ipsilateral MES broken down by protection stages and carotid revascularization procedures.

**Groups** **Protection stage**	**CEA (n = 17)**					**CAS (n = 16)**					**p-value** *
	**Mean**	**SD**	**Min**	**Med**	**Max**	**Mean**	**SD**	**Min**	**Med**	**Max**	
Pre-protection	51.6	146	0	13	615	291.8	275.1	16	210	1125	<0.0001
During protection	16.6	23	0	8	74	230.1	125.1	99	167,5	494	<0.0001
Post-protection	22.2	18.9	2	15	60	75.6	80.8	0	53	312	0.009
Total†	89.8	171.4	5	46	745	597.5	343.3	172	610.5	1640	<0.0001

CAS: carotid artery stenting; CEA: carotid endarterectomy; SD: standard deviation.

* Comparison between groups using the ANOVA test;

† Comparison between the protection stages using the ANOVA test: for the CEA group, there was no statistical difference between the stages; for the CAS group, p < 0.0001 for pre-protection versus post-protection, and for during protection versus post-protection, but no statistical difference between pre-protection and during protection.

**Figure 2:**
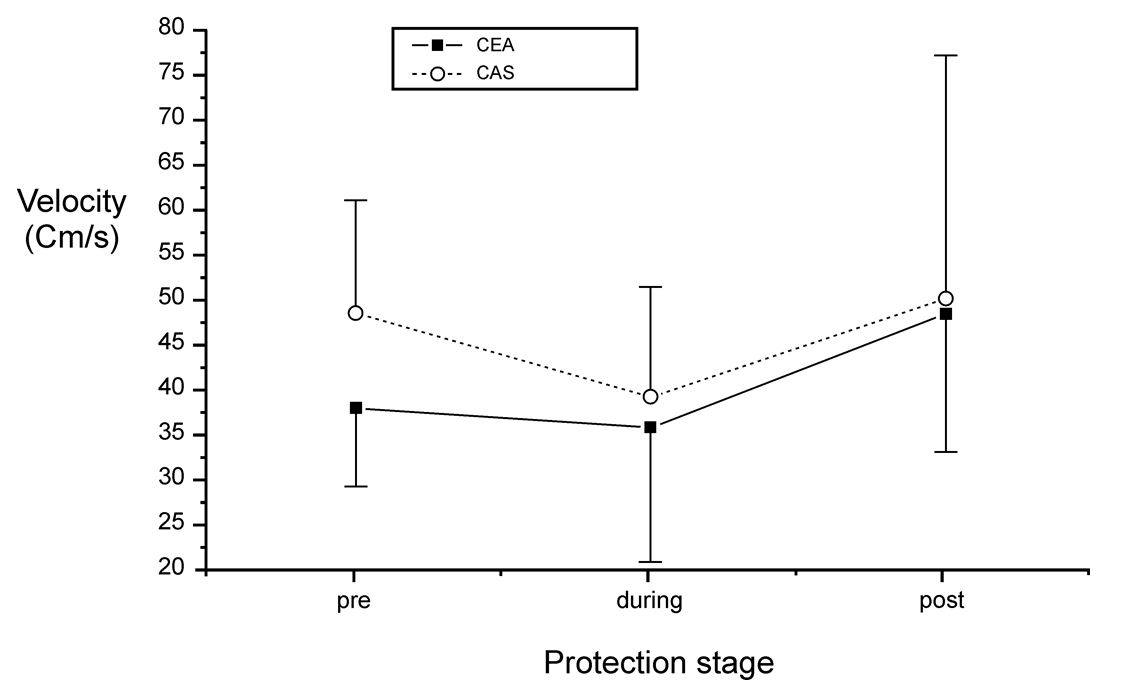
Average values with standard deviation of the mean velocities in the ipsilateral MCA in each protection stage.

Another important group of variables analyzed is the averages for mean blood flow velocities in the ipsilateral MCA (Table 4 and Figure 2). It is important to note that the variations in the average velocities were similar in both groups (p = 0.152). Furthermore, in both groups, the mean blood flow velocity within the MCA fell after the transition from pre-protection to during protection, and rose once again during post-protection, attaining values that were higher than both preceding stages (p <0.0001).

**Table 4:** Analysis of the averages of mean velocities of blood flow (cm/s) in the ipsilateral MCA, broken down by protection stages and carotid revascularization procedures.

**Groups** **Protection stage**	**CEA (n = 17)**					**CAS (n = 16)**					**p-value** *
	**Mean**	**SD**	**Min**	**Med**	**Max**	**Mean**	**SD**	**Min**	**Med**	**Max**	
Pre-protection	37.9	8.7	21	36	55	48.4	12.5	31	45.5	78	0.152
During protection	35.7	14.9	11	34	69	39.1	12.2	24.1	38.2	72.5	0.152
Post-protection	48.3	15.3	27	45	85	50	27	0.8	48.5	110	0.152
p-value†	<0.0001					<0.0001					-

CAS: carotid artery stenting; CEA: carotid endarterectomy.

* Comparison between groups using the ANOVA test;

† Comparison between protection stages using the ANOVA test.

## DISCUSSION

The correlation between microembolization detected by TCD during CEA and cerebral ischemia was demonstrated in 1994.^20^ According to one important study,^19^ both gaseous and solid emboli can cause cerebral injuries during carotid revascularization. In the present study, this distinction was not made. Another relevant feature of this study relates to counting microemboli and the manner in which noise related to contrast injection and electrical interference from the electric scalpel was excluded. A previous study has suggested it is necessary to discard these data to avoid methodological errors, but the study authors did not provide details on how to do this.^9^ These recommendations were followed in the present study. However, it was noted that the number of MES rose after contrast injection and during use of the electric scalpel, but did not sink back to baseline levels after these phases were over. These extra signals were not discarded in the present study and it is possible that these peculiarities are linked to the observation of a higher number of MES, in comparison to other studies.^4^^,^ 9 ^,^ 17 ^,^ 19

Given the sample size studied, it was not possible to relate microemboli to morbid events. However, it was demonstrated that MES were significantly more prevalent in patients treated with CAS, and this phenomenon was observed in all three stages of the procedure. Some studies have reported the same finding.^4^^,^ 9 ^,^ 17 ^,^ 19 The present study also found that the temporal distribution of microemboli was different in each group. In the CEA group, there was no statistical difference in the distribution of MES across the three stages, but in the CAS group, MES were most prevalent in the pre-protection and during protection stages.

To a certain extent, the results for CEA, contradict the findings of other authors^21^ who demonstrated differences in the temporal distribution of microemboli. They state that distal control of the internal carotid and installation of the shunt, both crucial moments during CEA, would result in more embolization.^21^ However, there are two aspects that differentiate the present study from that one: 1) in the present study, the technique used for endarterectomy (semi-eversion) results in less distal exposure and manipulation of the internal carotid artery; 2) it was not necessary to use a shunt in any of the cases in the present study.

On the other hand, MES noted during clamping could be explained by possible contralateral microemboli and intracranial atheroemboli. Furthermore, MES were undoubtedly more common when the internal and external carotid arteries were unclamped, while the common carotid artery was still clamped (see Methods section).

The data observed for the CAS group demonstrated a tendency to greater occurrence of MES in the pre-protection stage, although there was no statistical difference in relation to the second stage. It was clear that it was only once the stent was correctly positioned and arterial manipulation ceased, in the post-protection stage, that a significant drop in the number of MES occurred. Furthermore, it could be argued that the initial manipulation of the aortic arch and manipulation of the carotid lesion without any protection generate microemboli and that even once the distal filter has been installed protection is still incomplete since debris can escape through the filter or around the filter, which has been documented.^22^

Given that many neurological events can occur after removal of the brain-protection device, it can be assumed that a large number of carotid lesions treated with CAS continue to release embolic material after carotid intervention.^23^^,^ 24 These authors suggest that using closed-cell stents results in a significant decrease in post-procedural neurological events. However, two more recent studies^25^^,^ 26 found no difference between groups treated using closed and open-cell stents. The results of these more recent studies should avert any criticism of the exclusive use of open-cell stents in the present study.

The increase in velocity of blood flow through the MCA after carotid revascularization has been investigated by several authors.^27^^,^ 28 As was to be expected, the average blood flow velocity in the ipsilateral middle cerebral artery increased after all procedures.

Another important point is the reduction in average velocities during protection, observed in both groups, notwithstanding that this did not necessarily mean that the patient reached conceptual ischemia (< 30% of baseline). Other authors have observed several cases of this reduction in average blood flow velocity during CEA procedures, but in that study only 16 out of 49 patients exhibited a drop in blood velocity resulting in ischemia.^29^ It is relevant to note that while the CEA group in the present study did not have any cases of conceptual ischemia (< 30% of baseline), there was one case in the CAS group in which ischemia occurred for 312 seconds. However, there is a difference between this study and the one cited above,^29^ in that it defined ischemia as the period of time during which blood flow fell below 50% of the baseline blood flow, rather than 30%.

In the present study there was a similarity between the groups in terms of how the averages of the mean velocities decreased in the second stage, which was expected for CEA, but went against the initial hypothesis that CAS with a distal filter would not disrupt anterograde blood flow. In addition to saturation of the filter, there are other factors that contributed to this reduction, such as placement of the stent within the residual lumen of the internal carotid and the later balloon expansion of the stent.

In conclusion, in the light of TCD findings, CEA (semi-eversion technique) resulted in a lower incidence of MES than CAS with a distal filter in all protection stages, with a uniform temporal distribution in the CEA group and greater occurrence of MES in the first two stages in the CAS group. The averages of the mean velocity of flow within the MCA behaved similarly in both groups: the averages of the mean velocities tended to fall from the first to the second stage, and then rise from the second to the third, reaching levels higher than those in the first stage.
